# Divergent evolution of rice blast resistance *Pi54* locus in the genus *Oryza*

**DOI:** 10.1186/s12284-018-0256-8

**Published:** 2018-12-05

**Authors:** Lin Zhang, Yusuke Nakagomi, Takashi Endo, Mika Teranishi, Jun Hidema, Shusei Sato, Atsushi Higashitani

**Affiliations:** 1 Graduate School of Life Sciences, Tohoku University, Sendai, 980-8577 Japan; 2 Miyagi Prefectural Furukawa Agricultural Experiment Station, Osaki, 989-6227 Japan

**Keywords:** *Oryza*, *Pi54* alleles, Resistance gene, Blast disease, Evolution, Rice breeding

## Abstract

**Background:**

The rice blast resistance gene *Pi54* was cloned from *Oryza sativa ssp. indica* cv. Tetep, which conferred broad-spectrum resistance against *Magnaporthe oryzae*. *Pi54* allelic variants have been identified in not only domesticates but also wild rice species, but the majority of *japonica* and some *indica* cultivars lost the function.

**Results:**

We here found that *Pi54* (Os11g0639100) and its homolog Os11g0640600 (named as #11) were closely located on a 25 kbp region in *japonica* cv. Sasanishiki compared to a 99 kbp region in *japonica* cv. Nipponbare. Sasanishiki lost at least six genes containing one other R-gene cluster (Os11g0639600, Os11g0640000, and Os11g0640300). Eight AA-genome species including five wild rice species were classified into either Nipponbare or Sasanishiki type. The BB-genome wild rice species *O. punctata* was Sasanishiki type. The FF-genome wild rice species *O. brachyantha* (the basal lineage of *Oryza*) was neither, because *Pi54* was absent and the orientation of the R-gene cluster was reversed in comparison with Nipponbare-type species. The phylogenetic analysis showed that #11gene of *O. brachyantha* was on the root of both *Pi54* and #11 alleles. All Nipponbare-type *Pi54* alleles were specifically disrupted by 143 and 37/44 bp insertions compared to Tetep and Sasanishiki type. In addition, *Pi54* of *japonica* cv. Sasanishiki lost nucleotide-binding site and leucine-rich repeat (NBS–LRR) domains owing to additional mutations.

**Conclusions:**

These results suggest that *Pi54* might be derived from a tandem duplication of the ancestor #11 gene in progenitor FF-genome species. Two divergent structures of *Pi54* locus caused by a mobile unit containing the nearby R-gene cluster could be developed before domestication. This study provides a potential genetic resource of rice breeding for blast resistance in modern cultivars sustainability.

**Electronic supplementary material:**

The online version of this article (10.1186/s12284-018-0256-8) contains supplementary material, which is available to authorized users.

## Background

*Oryza sativa*, including two major subspecies *japonica* and *indica*, is the staple food for half the world and of pivotal importance in worldwide food production and security (Chang 1976; Lu 1999; Ammiraju et al. 2006). It is estimated that we need to produce 60% more rice between 2010 and 2050 to meet increasing demand (Alexandratos and Bruinsma 2012; Saito et al. 2017). Plant diseases are threatening the crops productions worldwide. For example, rice blast disease, caused by the fungus *Magnaporthe oryzae* (*M. oryzae*), is one of the most damaging rice diseases, and rice yield loss was up to 100% (Liu et al. 2014). Plants have evolved sophisticated defense response to resist pathogens infection over the course of evolution. The first layer of plant defense is pathogen-associated molecular patterns triggered immunity, and the second layer is effector-triggered immunity, which is mediated by plant resistance genes (R-genes) (Dangl et al. 2013). Most R-genes encode proteins with nucleotide-binding site and leucine-rich repeats (NBS–LRR) domains (Gay et al. 1991; Hammond-Kosack and Jones 1997; Song et al. 1997; Kobe and Kajava 2001; McHale et al. 2006). Multiple R-genes are located in clusters and all these clusters were abundant on chromosome 11 of rice genome (Zhou et al. 2004; Yang et al. 2006; Zhang et al. 2014; Vasudevan et al. 2015; Ashkani et al. 2016).

In recent rice-breeding programs, pyramiding of R-genes has been an effective strategy for achieving durable resistance in commercial crops (Ashkani et al. 2016; Xiao et al. 2017). Until now, over 100 major blast R-genes against *M. oryzae* have been identified, but only 30 of them have been cloned and characterized (Wang et al. 2017). Nearly all of the cloned R-genes encodes NBS–LRR proteins except *Pid2*, which encodes a receptor-like kinase (Chen et al. 2006). Some R-genes against rice blast disease such as *Pi1, Piz-5*, *Pita*, and *Pi5*, have been introgressed into agronomically superior rice cultivars by marker-assisted selection (Hittalmani et al. 2000; Narayanan et al. 2002; Liu et al. 2003; Lee et al. 2009). Marker-assisted backcrossing has been used with the blast resistance genes *Piz-5* and *Pi54* to develop improved restorers, Pusa1602 (with *Piz-5*) and Pusa1603 (with *Pi54*) (Singh et al. 2012).

The blast resistance gene *Pi54* (also known as *Pi-k*^*h*^), encoding an NBS–LRR protein, was initially identified and cloned from the *indica* cv. Tetep; it confers broad-spectrum resistance against Indian rice blast isolates (Rai et al. 2011). Nowadays, many blast resistance alleles of *Pi54* were also cloned from various wild rice species providing a high degree of resistance to *M. oryzae* (Das et al. 2012; Devanna et al. 2014). Moreover, *Pi54* allelic variants, including resistant and susceptible genotype, have been detected through sequence-based allele mining. The studies revealed that the numbers of coding DNA sequences (CDSs) at the *Pi54* varies from 0 to 3 and the predicted proteins consist of 73 to 486 amino acid (AA) residues (Thakur et al. 2015). Specifically, the LRR domain showed a high level nucleotide variation and the selection pressure was high in this domain (Kumari et al. 2013; Thakur et al. 2015). The NBS domain of *Pi54* alleles also revealed diversity with amino acid sequence polymorphism (Kumari et al. 2013). However, it is still unclear how *Pi54* and its locus diversity has evolved and spread in genus *Oryza*.

Recent *Oryza* genome projects have clarified the domestication history of rice (Zhang et al. 2014; Stein et al. 2018). The 27 Oryza species are divided into 11 genome types, 6 of which are diploid (*n* = 12: AA, BB, CC, EE, FF and GG) and 5 of which are polyploid (*n* = 24: BBCC, CCDD, HHJJ, HHKK and KKLL). Two species, *O. sativa* in Asia ~ 10,000 years ago and *O. glaberrima* in Africa ~ 3000 years ago, have been independently domesticated as rice. Cultivated rice belongs to the AA genome group. Here, we report two considerably divergent structures of the *Pi54* gene and its locus in two *O. sativa* ssp*. japonica* domesticated cultivars Nipponbare and Sasanishiki. We compared these structures with those in six AA-genome species (*O. nivara, O. glumaepatula*, *O. barthii, O. glaberrima, O. rufipogon,* and *O. meridionalis*), one BB-genome species *O. punctata*, and one FF-genome species *O. brachyantha* including not only domesticated rice but also wild species. Our analysis revealed origin and evolution of the *Pi54* gene and its locus in genus *Oryza* and provided potential genetic resources for breeding for rice blast resistance in modern cultivars.

## Methods

### Plant materials, growth conditions, DNA extraction, and genomic information

*Oryza sativa ssp. japonica* cultivars, including Hitomebore, Nipponbare, and Sasanishiki, were grown in pots and kept in a growth chamber at 30 °C during the day and 22 °C at night with a 12-h photoperiod after 3 days germinating at 30 °C. DNA was extracted from fresh leaves using a modified cetyl trimethylammonium bromide (CTAB) protocol (Teranishi et al. 2004). The genomic information of Nipponbare, *O. glumaepatula* (GEN1233_2), *O. nivara* (IRGC100897), *O. barthii* (IRGC105608), *O. glaberrima* (IRGC:96717), *O. rufipogon* (W1943), *O. meridionalis* (W2112), *O. punctata* (IRGC105690), *O. brachyantha* (IRGC101232), and *O. sativa* ssp. *indica* (9311) was downloaded from EnsemblPlants (http://plants.ensembl.org/index.html). The genomic information of *Pi54* reference allele of Tetep is available from GenBank with accession number CCD33085.

### PCR analyses

A primer set *Pi54* MAS (Additional file 1: Table S1) (Ramkumar et al. 2010) was used to identify the difference of *Pi54* allele between Nipponbare, Sasanishiki and Hitomebore. To detect the presence or absence of genes in *Pi54* locus, in total 14 genes based on the Os-Nipponbare-Reference-IRGSP-1.0 reference genome (International Rice Genome Sequencing Project, IRGSP), primer sets were prepared and listed in Additional file 1: Table S1. The 14 genes were named as #1 to #14. To identity Nipponbare and Sasanishiki-type *Pi54* locus in 24 modern *japonica* cultivars (including Nipponbare, Sasanishiki, and Hitomebore), specific PCR primer sets were designed with two ways, either according to left-side and right-side border sequences of the divergent region of *Pi54* locus or Sasanishiki-specific polymorphisms of *Pi54* and #11 genes. The primer sequences were listed in Additional file 1: Table S2. All the primers were designed in Primer3Plus software (Untergasser et al. 2007). PCR condition was using the following temperature profile: initial DNA denaturation, 94 °C for 3 min; followed by 35 cycles of denaturation, 98 °C for 10s; annealing, 68 °C for 15 s; extension, 68 °C for 30s or 1 min (according to the product size); and final extension at 72 °C for 5 min and then hold at 4 °C using PrimerSTAR GXL DNA polymerase (Takara, Japan).

### Sequence analysis of the *Pi54* locus of Sasanishiki using a BAC clone library

A high-density Sasanishiki bacterial artificial chromosome (BAC) library in the plndigoBAC-5 (Takano et al. 2013) was used. A BAC clone harboring gene loci from Os11g0638700 (named as #1) to Os11g0641300 (named as #14) was obtained and filtered using the primer sets Os11g0639000 (named as #3) and Os11g0640800 (named as #12) (Additional file 1: Table S1). The sequencing primers for confirming exact location of the inserted fragment in BAC clone were pIB FP and pIB RP. The selected BAC clone was used to extract the plasmid in a large-construct DNA purification kit (NucleoBond Xtra BAC). The position of the inserted Sasanishiki fragment is 25,220,854 to 25,448,655 bp on chromosome 11 according to the reference genome; it harbors the genes from #1 (25,237,345 to 25,239,585) to #14 (25,399,957 to 25,400,721). The region containing *Pi54* (#4)–#12 was amplified from this clone by long-range PCR. The obtained PCR product was subcloned and sequenced by ABI3130 Genetic Analyzer with a BigDye Terminator Sequence Ready kit (Applied Biosystems, http://www.appliedbiosystems.com). Sequencing primers are listed in Additional file 1: Tables S3 and S4.

Multiple sequence alignments were generated in GenomeMatcher (Ohtsubo et al. 2008) and CLUSTALX2 (Larkin et al. 2007) software. Gene loci of the sequenced fragment was predicted by Augustus software (http://bioinf.uni-greifswald.de/augustus/submission.php). Sequence similarity analysis was performed with the ncbi-blast-2.6.0 tool (ftp://ftp.ncbi.nlm.nih.gov/blast/executables/blast+/LATEST). The threshold expectation value was set to 10^− 4^, which was determined empirically to filter out most of the spurious hits. The translated AA sequence of each of the predicted genes of Sasanishiki and of each of genes in Nipponbare (Os11g0638700 to Os11g0641300) was used as a query against the genome of the other accessions that was downloaded from EnsemblPlants.

### Structural and phylogenetic analyses of the homologous genes *Pi54* and #11

The protein sequences encoded by two predicted Sasanishiki genes were acquired in Augustus. The NBS and LRR domains were searched using the CDART (https://www.ncbi.nlm.nih.gov/Structure/lexington/lexington.cgi?cmd=rps) and Pfam (http://pfam.xfam.org/) tools. The coiled-coil (CC) domain was predicted using the COILS server (http://embnet.vital-it.ch/software/COILS_form.html). Repetitive elements were analyzed using the RepeatMasker Web Server (http://www.repeatmasker.org/cgi-bin/WEBRepeatMasker). Information on transposable elements was downloaded from the Repbase database (http://www.girinst.org/repbase/).

To assess the evolutionary relationship of the conserved DNA sequences of alleles *Pi54* and #11, molecular phylogenetic analysis was performed by the Maximum Likelihood method (ML) based on the JTT matrix-based model (Jones et al. 1992) in MEGA 7 software (Kumar et al. 2016). DNA sequences (21 in total) from Nipponbare, Sasanishiki, *O. glumaepatula*, *O. nivara*, *O. barthii*, *O. glaberrima*, *O .meridionalis*, *O. rufipogon*, *O. punctata*, *O. brachyantha* and *O. sativa* ssp. *indica* 9311 and Tetep were used.

## Results

### Divergent structures of the *Pi54* locus in *O. sativa* ssp. *japonica* cultivars

**Fig. 1:**
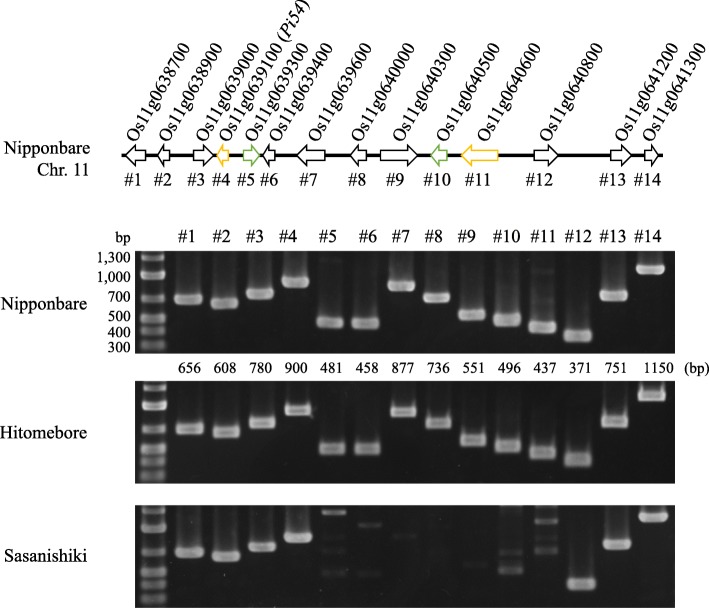
PCR analysis of 14 genes (Os11g0638700–Os11g0641300) in Nipponbare, Hitomebore and Sasanishiki. Marker fragment sizes are shown on the left PCR product sizes are shown at the bottom of the Nipponbare pane

A PCR-based co-dominant molecular marker, *Pi54* MAS, has been developed targeting a 144 bp insertion/deletion polymorphism in the exon of *Pi54* gene (Ramkumar et al. 2010). *Pi54* MAS is able to distinguish resistant (without 144 bp insertion) and susceptible genotype (with 144 bp insertion). To observe the *Pi54* alleles (Os11g0639100: named as #4) among *O. sativa* ssp*. japonica* cv. Nipponbare, Sasanishiki, and Hitomebore, we performed PCR amplification with *Pi54* MAS using genomic DNA. The results clearly indicated that Nipponbare and Hitomebore harbored the insertion but no such insertion was found in Sasanishiki (Additional file 1: Figure S1). To analyze the *Pi54* locus in Sasanishiki, we designed 14 primer sets to detect genes Os11g0638700 (#1) to Os11g0641300 (#14) in Nipponbare, Sasanishiki, and Hitomebore genome (Materials and method; Additional file 1: Table S1). All expected fragments were amplified successfully from the Nipponbare and Hitomebore genomes, but specific amplifications of genes from Os11g0639300 (#5) to Os11g0640600 (#11) failed in Sasanishiki genome (Fig. 1). To obtain the genomic fragment of Sasanishiki *Pi54* locus, we performed long-range PCR with the forward primer of *Pi54* and the reversed primer of #12 (Additional file 1: Table S1), but PCR amplification failed using Sasanishiki genomic DNA directly. Therefore, we screened a 23 K BAC clone library of the Sasanishiki genome to analyze the structure of the *Pi54* locus in Sasanishiki (Takano et al. 2013). We finally isolated a positive clone (plate No. 48, P12), sequenced it and found that this 25,061 bp nucleotide fragment contained 4 genes (*Pi54*, an unknown gene, #11, and #12; Sasanishiki type), while the 98,798 bp Nipponbare fragment included 9 genes (Nipponbare type) (Additional file 1: Figure S2). The genes from #5 to Os11g0640500 (#10) were lost in Sasanishiki. The genes #1, Os11g0638900 (#2), Os11g0639000 (#3), Os11g0641200 (#13), and #14 of Sasanishiki were more than 98% identical to those of Nipponbare, while *Pi54*, #11, and #12 were less than 93% identical (Table 1). The unknown gene found between *Pi54* and #11 in Sasanishiki was absent in the Nipponbare genome.

**Table 1:** Comparison of the sequences of genes Os11g0638700 to Os11g0641300 between Nipponbare and Sasanishiki

						Sequence alignments			
Nipponbare			Sasanishiki			NT		AA	
Gene_list	NT^a^	AA^b^	Gene_list	NT	AA	E-values ^c^	Identities	E-values	Identities
Os11g0638700 (#1)	2241	746	#1	2260	648	0	2237/2241 (99%)	0	648/712 (91%)
Os11g0638900 (#2)	1835	180	#2	1835	180	0	1833/1835 (99%)	4E-136	180/180 (100%)
Os11g0639000 (#3)	4349	173	#3	4268	222	0	2882/2933 (98%)	4E-088	124/151 (82%)
Os11g0639100 (#4)	941	112	#4	3735	920	0	864/939 (92%)	1E-049	88/100 (88%)
–	–	–	Unknown	2659	281	–	–	–	–
Os11g0639300 (#5)	2276	726	–	–	–	–	–	–	–
Os11g0639400 (#6)	722	107	–	–	–	–	–	–	–
Os11g0639600 (#7)	4823	935	–	–	–	–	–	–	–
Os11g0640000 (#8)	1947	648	–	–	–	–	–	–	–
Os11g0640300 (#9)	4157	1118	–	–	–	–	–	–	–
Os11g0640500 (#10)	2196	731	–	–	–	–	–	–	–
Os11g0640600 (#11)	6464	1101	#11	8509	924	0	2593/3179 (82%)	8E-122	224/410 (55%)
Os11g0640800 (#12)	3842	620	#12	2086	623	0	1720/1843 (93%)	0	552/620 (89%)
Os11g0641200 (#13)	5361	358	#13	5313	616	0	5271/5301 (99%)	0	306/353 (87%)
Os11g0641300 (#14)	765	236	#14	913	236	0	764/765 (99%)	1E-174	236/236 (100%)

^a^NT, nucleotide sequence

^b^AA, amino acid sequence

^c^E-values, expectation values

**Fig. 2:**
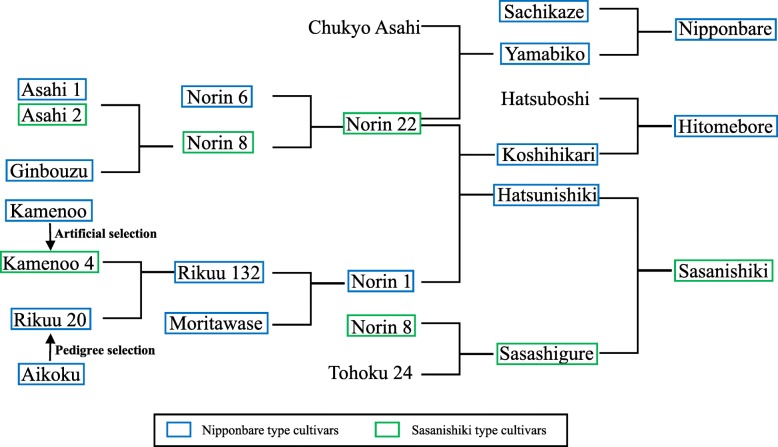
The breeding history of modern *O. sativa* ssp. *japonica* cultivars Nipponbare, Hitomebore, and Sasanishiki was obtained from Agriknowledge database (https://agriknowledge.affrc.go.jp/). The modern rice breeding in Japan was from Norin cultivars

To analyze the breeding history of the *Pi54* locus, we designed specific PCR primer sets to amplify the upstream and downstream border sites and found that the divergent structures of the *Pi54* locus are randomly distributed in the parental lines of Nipponbare, Sasanishiki, and Hitomebore (Additional file 1: Figure. S3, Fig. 2). The modern rice breeding in Japan was from Norin cultivars. Intriguingly, both types were found in early lines (Asahi 1 and Asahi 2, and Kamenoo, and Kamenoo 4) established before modern breeding more than 100 years ago (Fig. 2). These two types were also found in *japonica* rice cultivated in China with Hokushitami and Zaijian being the Sasanishiki type and Hexi 23 the Nipponbare type. These results suggest that each type of the *Pi54* locus was stably and widely spread among *japonica* cultivars before modern artificial breeding history.

### *Pi54* locus in the genus *Oryza*

**Fig. 3:**
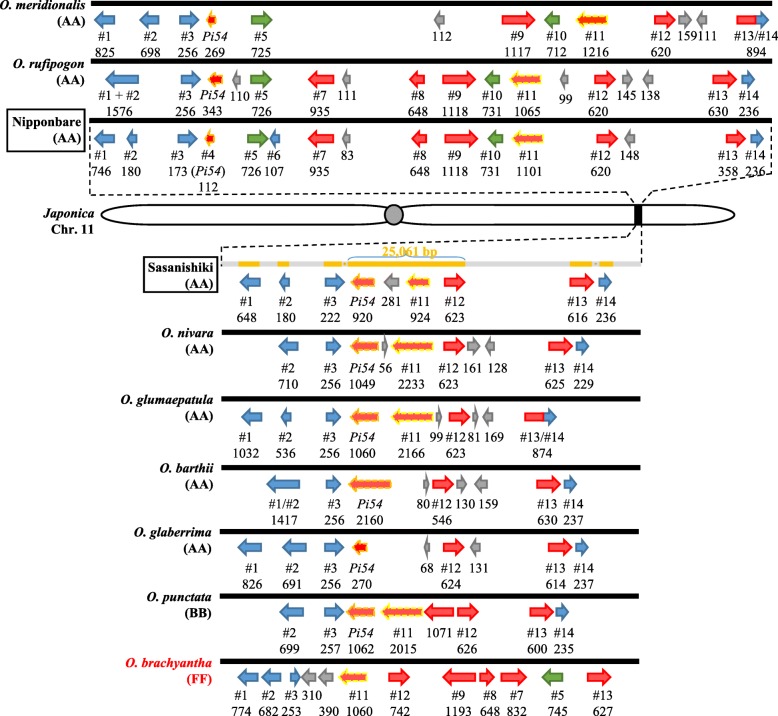
Genes homologous to #1 to #14. Nipponbare: the region corresponding to 25,237,345–25,400,721 bp on chromosome 11 of Os-Nipponbare-Reference-IRGSP-1.0. Arrows: red, NBS–LRR family-like genes; blue, miscellaneous genes; gray, presumed genes; green, translocated genes. Arrows with orange and/or yellow dashed (alleles of the *Pi54* and #11, respectively) line show genes that were used to conduct phylogenetic tree. Protein size (AA) is indicated under each gene ID*.* Gray bars in Sasanishiki correspond to unsequenced regions

**Fig. 4:**
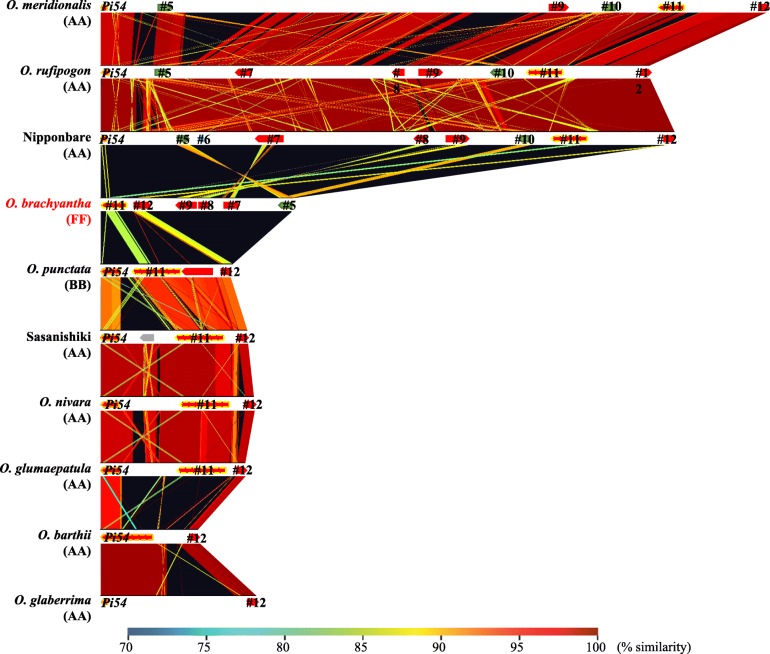
Multiple sequence alignment of divergent structures in the *Pi54* locus. DNA sequences from *Pi54* to #12 in 10 species were extracted and analyzed in GenomeMatcher software (Ohtsubo et al. 2008). The corresponding region in the reference genome Os-Nipponbare-Reference-IRGSP-1.0 is 25,263,336–25,362,133 bp on chromosome 11. Red arrows represent gene loci

Next, we investigated whether the two types of the *Pi54* locus are conserved. The *Pi54* locus of six AA-genome species was Nipponbare type in *O. rufipogon* and *O. meridionalis* and Sasanishiki type in *O. nivara*, *O. glumaepatula*, *O. barthi*, and *O. glaberrima* (Fig. 3). The synteny from *Pi54* to #12 was conserved within the types and the synteny of both flanking regions (#1 to *Pi54* on the left side and #12 to #14 on the right side) was highly conserved in all AA-genome species (Fig. 3). A multiple alignment analysis of DNA sequences from *Pi54* to #12 showed more than 90% identity within each type but little identity between the Nipponbare and Sasanishiki types (Fig. 4). The Nipponbare type had a long insertion containing genes #5 to #10 in comparison with the Sasanishiki type. In the African wild rice *O. barthii* and *O. glaberrima,* as well as in Nipponbare type species *O. rufipogon* (Asian) and *O. meridionalis* (Australian)*,* different insertions and deletions were found (Fig. 4). Altogether, these results indicate that the two divergent *Pi54* loci developed before the establishment of AA genome species*.*

The structure of the *Pi54* locus in *O. punctata* (BB-genome) lacked the region from genes #5 to #10, and thus was Sasanishiki type (Figs. 3, and 4), whereas that in *O. brachyantha* (FF-genome) consisted of genes #1, #2, #3, #11, #12, #9, #8, #7, #5, and #13, but *Pi54* and #10 were absent. In comparison with other Nipponbare type species, the orientation of #9, #8, #7, and #5 in *O. brachyantha* was reversed (Figs. 3, and 4). Thus, in FF-genome species, the *Pi54* locus had characters of both the Sasanishiki (close location of #3 and #11) and Nipponbare type (presence of #5, #7, #8 and #9).

### Diversity of the homologous genes *Pi54* and #11

**Fig. 5:**
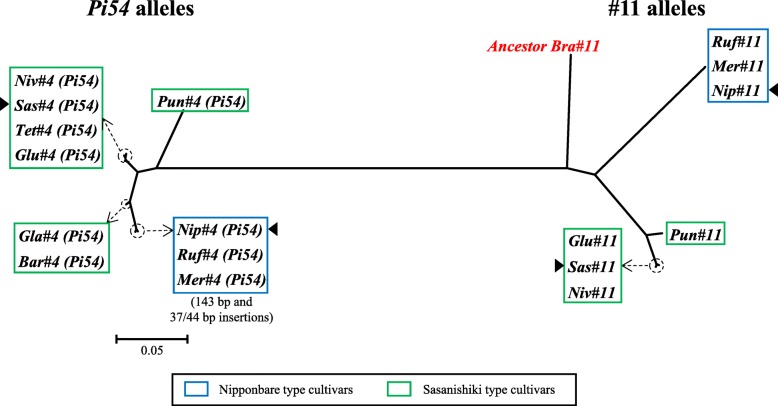
A maximum-likelihood phylogenetic tree showing relationships among the alleles of the *Pi54* and #11 genes. The tree contains 18 genomic DNA sequences both coding and non-coding. Sequences of 10 *Pi54* alleles were extracted according to the functional *Pi54* sequence in Tetep. Sequences of 8 alleles of #11 are complete coding DNA sequence (CDS). Nip, Nipponbare; Sas, Sasanishiki; Tet, Tetep; Glu, *O. glumaepatula*; Niv, *O. nivara*; Bar, *O. barthii*; Gla, *O. glaberrima*; Ruf, *O. rufipogon*; Mer, *O. meridionalis*; Pun, *O. punctata*; Bra, *O. brachyantha*. Locations of *Pi54* alleles on chromosome 11: Nip, 25,263,336–25,264,503; Ruf, 25,053,651–25,054,821; Mer, 21,747,946–21,749,123; Glu, 24,126,550–24,127,540; Niv, 22,154,478–22,155,468; Bar, 20,525,896–20,526,886; Gla, 18,706,523–18,707,513; Pun, 25,829,456–25,830,446. Locations of #11 alleles on chromosome 11: Nip, 25,338,884–25,345,347; Ruf, 25,125,041–25,131,339; Mer, 21,841,707–21,846,125; Glu, 24,139,300–24,147,809; Niv, 22,167,498–22,176,039; Bra, 14,455,855–14,459,981

In Nipponbare, in comparison with a 112 AA protein of gene *Pi54*, its homolog #11 (EnsemblPlants database) is a 1101 AA protein. Genome-wide blast analysis showed that all six AA-genome wild species analyzed carry orthologues of both *Pi54* and #11. Phylogenetic analysis showed that *Pi54* and #11 of Nipponbare were most closely related to those of Nipponbare type species *O. rufipogon* (Asian) and *O. meridionalis* (Australian) (Fig. 5). In contrast, *Pi54* and #11 of Sasanishiki were very similar to those of Sasanishiki type species, such as *O. nivara* (Asian) and *O. glumaepatula* (South American). The *Pi54* alleles of African *O. barthii* and *O. glaberrima* (Sasanishiki type species) was the sister group only of Nipponbare type species, and the lowest common ancestor of them was close to other Sasanishiki type species (such as *O. nivara* and *O. glumaepatula*).

We also performed phylogenetic analysis of *Pi54* and its homolog #11 with *O. punctata* (BB-genome) and *O. brachyantha* (FF-genome) that is placed in the basal lineage in *Oryza* (Chen et al. 2013) (Fig. 5). The divergence time between AA- and BB-genome species is estimated as more than 6 million years ago (Mya) and that between FF-and AA-BB genome species as 15 Mya (Stein et al. 2018). We found that #11 gene of *O. brachyantha* was on the root of both *Pi54* alleles and #11 alleles, while #11 of *O. punctata* was relatively close to Sasanishiki type species. In *O. brachyantha* (FF), *Pi54* was not detected in the expected region. In contrast, in *O. punctata* (BB), which was close to the root of all *Pi54* alleles, *Pi54* and an additional gene between #11 and #12 were found (Fig. 4). To sum up, we suggested that #11 of the FF-genome species was duplicated and *Pi54* originated from the duplicated before the divergence of FF- and AA-BB- genome species, and that this duplication was caused by natural rather than artificial selection.

**Fig. 6:**
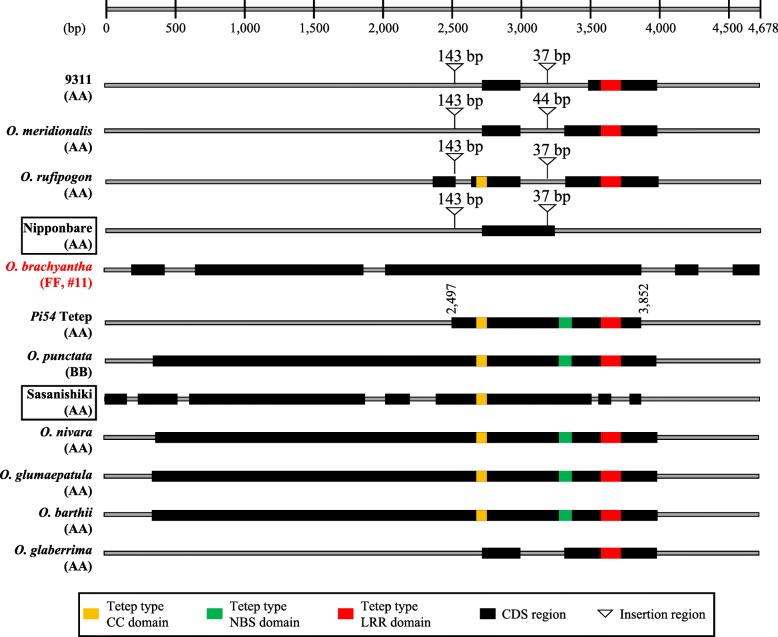
Schematic representation of DNA sequences of *Pi54* alleles. The sequences of Nipponbare, *O. rufipogon*, *O. meridionalis*, *O. sativa* ssp. *indica* (9311 and Tetep), and *O. glaberrima* include the gene sequences and 800 bp upstream sequences, while the other 5 sequences are gene sequences only (starting from ATG). CC, coiled coil

Insertions (143 bp and 37/44 bp) were found to be conserved in the *Pi54* alleles of 9311, *O. rufipogon* and *O. meridionalis* (Nipponbare type)*,* but not in Tetep, *O. nivara, O. glumaepatula*, *O. barthii*, or *O. glaberrima* (Sasanishiki type) (Fig. 6, Additional file 1: Figure S4). A 143 bp insertion is associated with susceptibility to blast disease (e.g., Nipponbare and Swarna), and resistant genotypes have no such insertion (e.g., Tetep and Suraksha) (Ramkumar et al. 2010). We found that the sequence of this insertion has high similarity to the non-autonomous DNA transposon Helitron-N91 (Additional file 1: Figure S5). The 37 or 44 bp insertion results in a loss of the NBS domain (Fig. 6). Sasanishiki lacks these insertions but has other mutations in the NBS and LRR domains, which may result in loss of *Pi54* function (Fig. 6).

### Interspersed repeats of the *Pi54* locus in *Oryza* species

To better understand the divergent structures of the *Pi54* locus, we analyzed interspersed repeat sequence in the region from *Pi54* to #12 (exception: from #11 to #5 of in *O. brachyantha*) (Table 2). All AA-genome species harbored a high level of interspersed repeats. For example, interspersed repeats occupied 55.64% of 99 kbp in Nipponbare and 33.43% of 25 kbp in Sasanishiki. In comparison with AA-genome species, the percentages of repetitive sequence of the ancestral species *O. brachyantha* (FF-genome, #11 to #5, 32 kbp) and *O. punctata* (BB-genome, *Pi54* to #12, 22 kbp) were significantly low, which occupied 10.90% and 3.17%, respectively. This high level of repetitive sequence in the *Pi54* locus of AA-genome species potentially lead to considerable diversity between Nipponbare and Sasanishiki type species. To further understand evolution of duplicated genes (#4 and #11; #5 and #10) in the *Pi54* locus, we analyzed transposons in the flanking regions of these duplicated genes in *O. brachyantha*, Nipponbare type species Nipponbare, *O. rufipogon*, and *O. meridionalis*, and Sasanishiki type species *O. nivara*. 26 non-autonomous transposons of both retroelements and DNA transposons were identified in total (Additional file 1: Table S5). In particular, the number of transposon that located on upstream of #5 is significantly higher than the other flanking regions of duplicated genes. Thus, the high level of interspersed repeats and a great number of transposons in the *Pi54* locus of AA-genome species may reflect underlying mechanisms of establishment of divergent structures and also increase genome instability.

**Table 2:** Interspersed repeat sequence analysis of divergent structures in the *Pi54* locus

Species	Length (bp)	Percentage of sequence			
		Retroelements	DNA transposons	Unclassified	Total interspersed repeats
*O. meridionalis* (AA)	112,929	9.34%	21.09%	6.81%	37.24%
*O. rufipogon* (AA)	92,702	13.45%	21.07%	18.34%	52.86%
Nipponbare (AA)	98,798	12.73%	21.35%	21.56%	55.64%
*O. brachyantha* (FF)	32,280	7.59%	2.37%	0.94%	10.90%
*O. punctata* (BB)	22,183	1.58%	1.59%	0.00%	3.17%
Sasanishiki (AA)	25,061	27.36%	5.99%	0.09%	33.43%
*O. nivara* (AA)	25,651	25.92%	5.11%	0.63%	31.66%
*O. glumaepatula* (AA)	22,674	18.87%	5.45%	0.10%	24.42%
*O. barthii* (AA)	16,743	0.00%	19.76%	1.84%	21.60%
*O. glaberrima* (AA)	26,686	6.76%	39.67%	2.13%	48.56%

## Discussion

### The evolutionary mechanisms of R-genes

The mechanism of interaction between host plants with R-genes encoding NBS–LRR protein and pathogen is explained by gene-for-gene resistance theory (Van Der Biezen and Jones 1998; McHale et al. 2006). R and *Avr* genes have co-evolved with natural selection in host–pathogen interactions (Sharma et al. 2012). Rapid evolution of the fungus *M. oryzae* occurs through non-synonymous variations, which frequently result in gain or loss of function of the *Avr* genes (Xue et al. 2012; Huang et al. 2014; Zhang et al. 2015). Resistant plants have acquired high levels of allelic diversity, new R-genes or copy number variations (CNVs) thereby increasing the durability of resistance (Yu et al. 2011; Jacob et al. 2013; Wang et al. 2014). In *Oryza*, the copy number of R-genes is markedly higher in both the *indica* and *japonica* cultivars than in wild rice, probably because of artificial selection for increased R-gene diversity and CNVs (Zhang et al. 2014; Stein et al. 2018). For example, 631 R-genes are predicted in the Nipponbare genome but only 307 in the genome of wild species *O. brachyantha* (Zhang et al. 2014; Zhu et al. 2014).

In this study, we found that an R-gene cluster located on the *Pi54* locus. In terms of *Pi54* gene of this locus, the family size is 2.8 ± 1.1 genes per species in 13 *Oryza* species*,* considerably smaller than the sizes of the *RGA4* (9.8 ± 3.1), *RGA5* (22.5 ± 5.2), *Pik1* (5.6 ± 1.6), and *Pik2* (18.7 ± 3.5) families (Stein et al. 2018). Suggested that *Pi54* family was conserved between modern and ancestral *Oryza* species with a small and stable gene family size. Therefore, to enhance the durability of rice blast resistance, *Pi54* may evolve through high levels of allelic diversity rather than CNVs. In terms of R-genes #7 to #9 that encode NBS–LRR proteins, the copy number of these three genes in Nipponbare is 36, 8, and 3, respectively, in contrast to 26, 5, and 4 in the progenitor *O. brachyantha* (EnsemblPlants database). Suggested that R-genes #7 to #9 might evolve with either high levels of allelic diversity or CNVs. To sum up, many R-genes could evolve with high levels of allelic diversity as *Pi54*. Thus, for *Pi54*-like R-genes, the study of exploring allelic diversity would be essential.

### An evolutionary model of two divergent structures of the *Pi54* locus

**Fig. 7:**
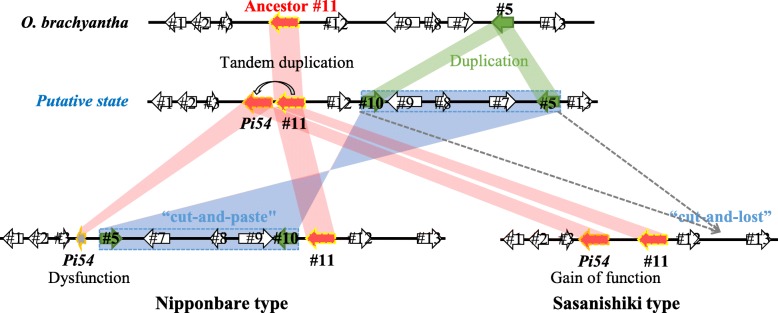
Model of the evolution of the two types of *Pi54* locus. In particular, the *Pi54* allele in Sasanishiki has lost its function because of several SNPs and small insertions and deletions. Dashed rectangle represents the mobile unit

To gain insights into evolution of the two divergent structures of the *Pi54* locus, we conducted genome-wide blast analysis (Fig. 3) and a phylogenetic analysis (Fig. 5) and deduced evolutionary history (Fig. 7). In the FF-genome species *O. brachyantha*, only #11 gene was localized in the *Pi54* locus but its homolog *Pi54* gene was undetectable in the whole genome. In addition, the phylogenetic analysis revealed that #11 gene of *O. brachyantha* was on the root of both *Pi54* and #11 alleles in other *Oryza* species. Taken together, we assumed that a tandem duplication of the ancestral #11 gene in progenitor FF-genome species led to the emergence of *Pi54*. Gene #5 with a domain of unknown function DUF594 was also duplicated, leading to the emergence of #10, which was then inserted in inverted orientation downstream of #9. The genes surrounded by homologous genes #5 and #10 might have become a mobile unit. In the Nipponbare type, this unit was integrated between *Pi54* and #11 in inverted orientation by “cut-and-paste” and might result in a shortening of the N-terminal portion of *Pi54* gene. As with the opposite orientation of the region from #5 to #10 (~ 60 kbp) in *O. brachyantha*, some other paracentric inversions have been reported between AA- and FF-genome species either (Stein et al. 2018). In the Sasanishiki type, this unit was “cut-and-lost” (Fig. 7). Besides, the *Pi54* locus of Nipponbare and Sasanishiki type were firstly observed in wild species *O. meridionalis* (AA-genome) and *O. punctata* (BB-genome), respectively. The mean AA-BB divergence time was 6.76 Mya, while the split time between *O. meridionalis* and other AA-genome species was 2.41 Mya (Stein et al. 2018). Along with domestication of *O. sativa* and *O. glaberrima* starting from ~ 10,000 and ~ 3000 years ago separately, these findings revealed that these two divergent *Pi54* loci had been developed before domestication. Taken together, in the evolutionary model, the duplication events are important factors in the generation of gain-of-function genes, including R genes (Hulbert et al. 2001; Leister 2004; Guo et al. 2011). The evolution of the two divergent structures of the *Pi54* locus revealed an ongoing birth and death process in R-genes.

Intriguingly, a similar unit (the same pattern as #5 and #10 with the orientation of genes toward each other and several genes between them) surrounded by OPUNC01G04010 and OPUNC01G04050 of the #5 gene family exists on chromosome 1 in the BB-genome species *O. punctata* (Additional file 1: Table S6). This unit is absent in several AA-genome species (*O. sativa* cv*.* Nipponbare, *O. rufipogon*, *O. meridionalis*, *O. nivara*, and *O. glumaepatula*), while the flanking sequences of this unit are well conserved. In the FF-genome species *O. brachyantha*, linkage of a single gene of the #5 gene family (OB01G13970) to the flanking sequences strongly supports our evolutionary model of the two divergent structures of the *Pi54* locus. In Nipponbare genome, the #5 gene family is expanded and 35 homologous genes are identified (EnsemblPlants database). It may contribute instability of rice genomes.

### Surviving genus *Oryza* with the divergent *Pi54* loci

*Pi54* alleles with high levels of tolerance to rice blast disease were identified and clone from not only domesticated but also wild rice species (Das et al. 2012; Devanna et al. 2014). In case of domesticated rice, it has been reported that the frequency of the functional *Pi54* genotype is higher in the *indica* subspecies (up to 87.9%) than in *javanica* (8.6%, now known as tropical *japonica*) and *japonica* (3.5%) (Vasudevan et al. 2015). Even in *japonica* cv. Sasanishiki that the nucleotide sequence of *Pi54* allele is similarity to *indica* cv. Tetep, a dysfunctional *Pi54* genotype appeared because of accumulation of polymorphisms and small insertions or deletions (Fig. 6). In case of wild rice species, we found that *Pi54* alleles in *O. rufipogon* and *O. meridionalis* (distributed in East Asia and Oceania (Stein et al. 2018)) were dysfunctional because of small (143 bp and 37/44 bp) insertions or a mobile unit surrounded by the homologous genes #5 and #10.

Recently, Zhong et al. (2018) found that the blast fungi *M. oryzae* were grouped into three major globally distributed clades. In the clade 2, most pathogens were isolated from *indica* rice-growing area. In the clade 3, most were isolated from *japonica* rice-growing area. Each clade shows different mating type (Zhong et al. 2018). Therefore, *Pi54* function may be essential for *indica* cultivars to resist the clade 2 pathogens but not for most *japonica* cultivars and several wild rice species, such as Nipponbare, *O. rufipogon*, and *O. meridionalis*. Taken together, we came up with a hypothesis of strategies of the divergent *Pi54* loci. Sasanishiki type species might evolve with the functional *Pi54* genotype at the cost of the loss of several NBS–LRR proteins (#7 to #9). In the Nipponbare type species, *Pi54* alleles became a pseudogene, and three R-genes (#7 to #9) accumulated in the adjacent region as compensation. Both type *Pi54* loci could confer genus *Oryza* with advantages surviving diverse pathogens distributed in different areas.

However, under global warming, pyramiding of the blast resistance gene *Pi54* into *japonica* commercial cultivars would be urgent for rice breeding programs, because the geographic range of pathogens interacted with *Pi54* alleles will expand owing to increasing temperatures (Stein et al. 2018). Our study of the *Pi54* locus in Nipponbare and Sasanishiki type species provides evolutionary insights into the generation of diversity and a potential genetic resource of rice breeding for blast resistance in modern cultivars sustainability.

## Conclusions

We found two divergent structures of *Pi54* locus that one carried dysfunctional *Pi54* gene but an R-gene cluster accumulated in the *Pi54* locus as compensation in an approximately 99 kbp region and was defined as Nipponbare type. Another one only harbored functional *Pi54* gene (except Sasanishiki) but at the cost of loss of the R-gene cluster in an around 25 kbp region and was considered as Sasanishiki type. Both of the divergent *Pi54* loci are widely distributed in modern *japonica* cultivars and wild rice species, including AA-, BB-, and FF-genome species. Furthermore, these two divergent loci had been developed before domestication and thus was caused by natural selection rather than artificial selection. Together with phylogenetic analysis, we came up with an evolutionary model of the two divergent structures of *Pi54* locus. This study could contribute to rice breeding programs and pave a novel way to understanding genetic evolution.

## Additional file

Additional file 1: **Figure S1.** Selection and identification of Pi54 alleles using primer set Pi54 MAS. **Figure S2.** Alignment of the divergent regions from Sasanishiki and Nipponbare. **Figure S3.** Detection of divergent structures at the *Pi54* locus in modern *O. sativa* cultivars. **Figure S4.** DNA sequences of *Pi54* alleles. **Figure S5.** Alignment of Helitron-N91 and the 143 bp insertion in Nipponbare. **Table S1.** Primer sets for PCR amplification. **Table S2.** Primer sets for identification of Nipponbare and Sasanishiki type species. **Table S3** Primers for sequencing. **Table S4.** Primers for sequencing the genes flanking the 25,061 bp region in Sasanishiki. **Accession codes**. Sequence data of Sasanishiki the *Pi54* locus. **Table S5.** Transposable elements in the flanking regions of *Pi54*, #5, #10 and #11. **Table S6.** Genes Orthologous to a mobile unit on chromosome 1 in *Oryza* species. (PDF 1280 kb)

## Abbreviations

AA: Amino acid
BAC: Bacterial artificial chromosome
CC: Coiled-coil domain
CDS: Coding DNA sequence
CNV: Copy number variation
CTAB: Cetyl trimethylammonium bromide
LRR: Leucine-rich repeats domain
NBS: Nucleotide-binding site domain
R-gene: Resistance gene
