# Supplementary material for: Divergent evolution of rice blast resistance Pi54 locus in the genus Oryza
Source: Rice (N Y). 2018 Dec 5;11:63. doi: 10.1186/s12284-018-0256-8 (PMC6281543; doi:10.1186/s12284-018-0256-8)
Supplement: Supplementary file 1 — Figure S1. Selection and identification of Pi54 alleles using primer set Pi54 MAS. Figure S2. Alignment of the divergent regions from Sasanishiki and Nipponbare. Figure S3. Detection of divergent structures at the Pi54 locus in modern O. sativa cultivars. Figure S4. DNA sequences of Pi54 alleles. Figure S5. Alignment of Helitron-N91 and the 143 bp insertion in Nipponbare. Table S1. Primer sets for PCR amplification. Table S2. Primer sets for identification of Nipponbare and Sasanishiki type species. Table S3 Primers for sequencing. Table S4. Primers for sequencing the genes flanking the 25,061 bp region in Sasanishiki. Accession codes. Sequence data of Sasanishiki the Pi54 locus. Table S5. Transposable elements in the flanking regions of Pi54, #5, #10 and #11. Table S6. Genes Orthologous to a mobile unit on chromosome 1 in Oryza species. (PDF 1280 kb) [file 12284_2018_256_MOESM1_ESM.pdf]

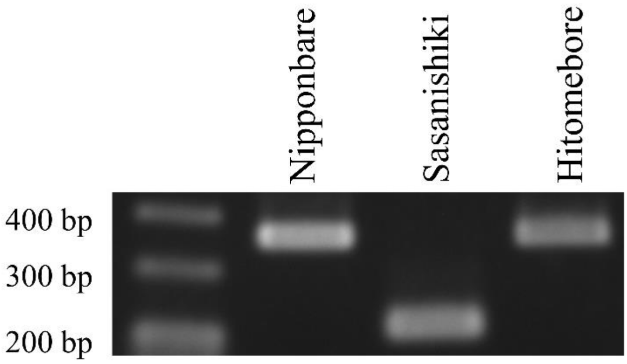

Figure S1 Selection and identification of *Pi54* alleles using primer set *Pi54* MAS. *Pi54* MAS was developed by Ramkumar et al. 2010 and could distinguish resistance and susceptible cultivars based on a 144 bp insertion in susceptible cultivars. The product sizes of resistance and susceptible cultivars are 215 and 358 bp, respectively.

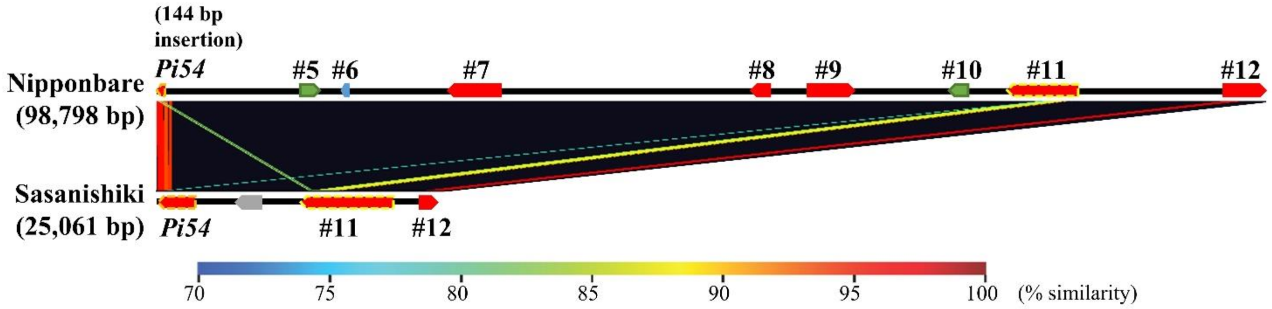

Figure S2 Alignment of the divergent regions from Sasanishiki and Nipponbare. Nipponbare: the region corresponding to 25,263,336–25,362,133 bp on chromosome 11 of Os-Nipponbare-Reference-IRGSP-1.0. The alignment was produced in GenomeMatcher softerware. Arrows: red, NBS–LRR family-like genes; blue, miscellaneous genes; gray, presumed genes; green, translocated genes

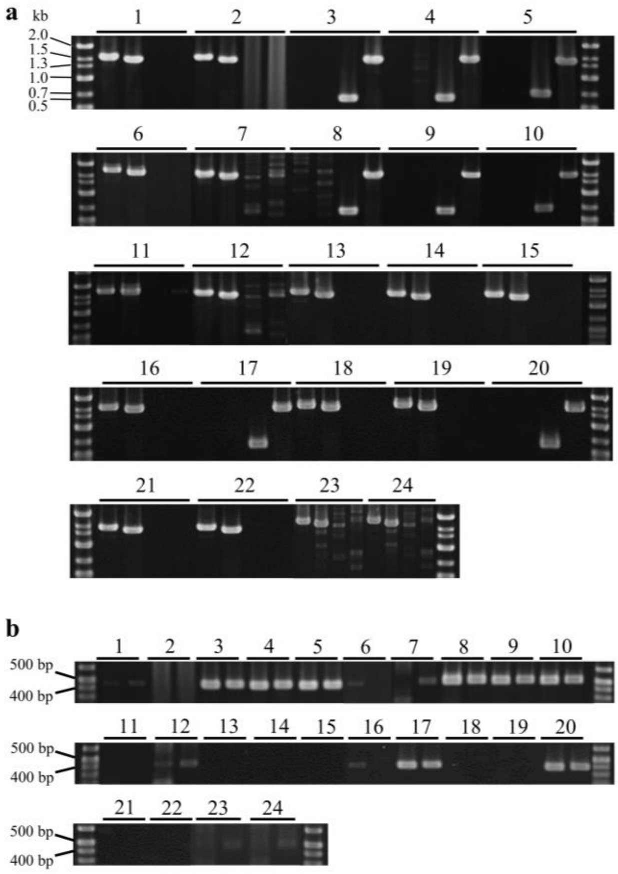

Figure S3 Detection of divergent structures at the *Pi54* locus in modern *O. sativa* cultivars. PCR fragments were separated in 1.5% agarose gel. (a) Detection of the locations of the left and right boundaries of the divergent region between Nipponbare and Sasanishiki in *Pi54* locus. The primer sets were Nipponbare type (N)-specific-left (1,570 bp), N-specific-right (1,491 bp), Sasanishiki type (S)-specific-left (619 bp), and S-specific-right (1,484 bp). (b) Detection of the *Pi54* and #11 genes of Sasanishiki type (450 bp and 468 bp fragment, respectively). 1, Norin 1; 2, Norin 6; 3, Norin 8; 4, Norin 22; 5, Asahi 2; 6, Hitomebore; 7, Nipponbare; 8, Sasanishiki; 9, Hokushitami (China); 10, Zaijian (China); 11, Hexi 23 (China); 12, Koshihikari; 13, Asahi 1; 14, Aikoku; 15, Moritawase; 16, Kamenoo; 17, Kmenoo 4; 18, Ginbouzu; 19, Hatsunishiki; 20, Sasashigure; 21, Rikuu 20; 22, Rikuu 132; 23, Yamabiko; 24, Sachikaze. Cultivars without geographic origin in parentheses are grown in Japan.

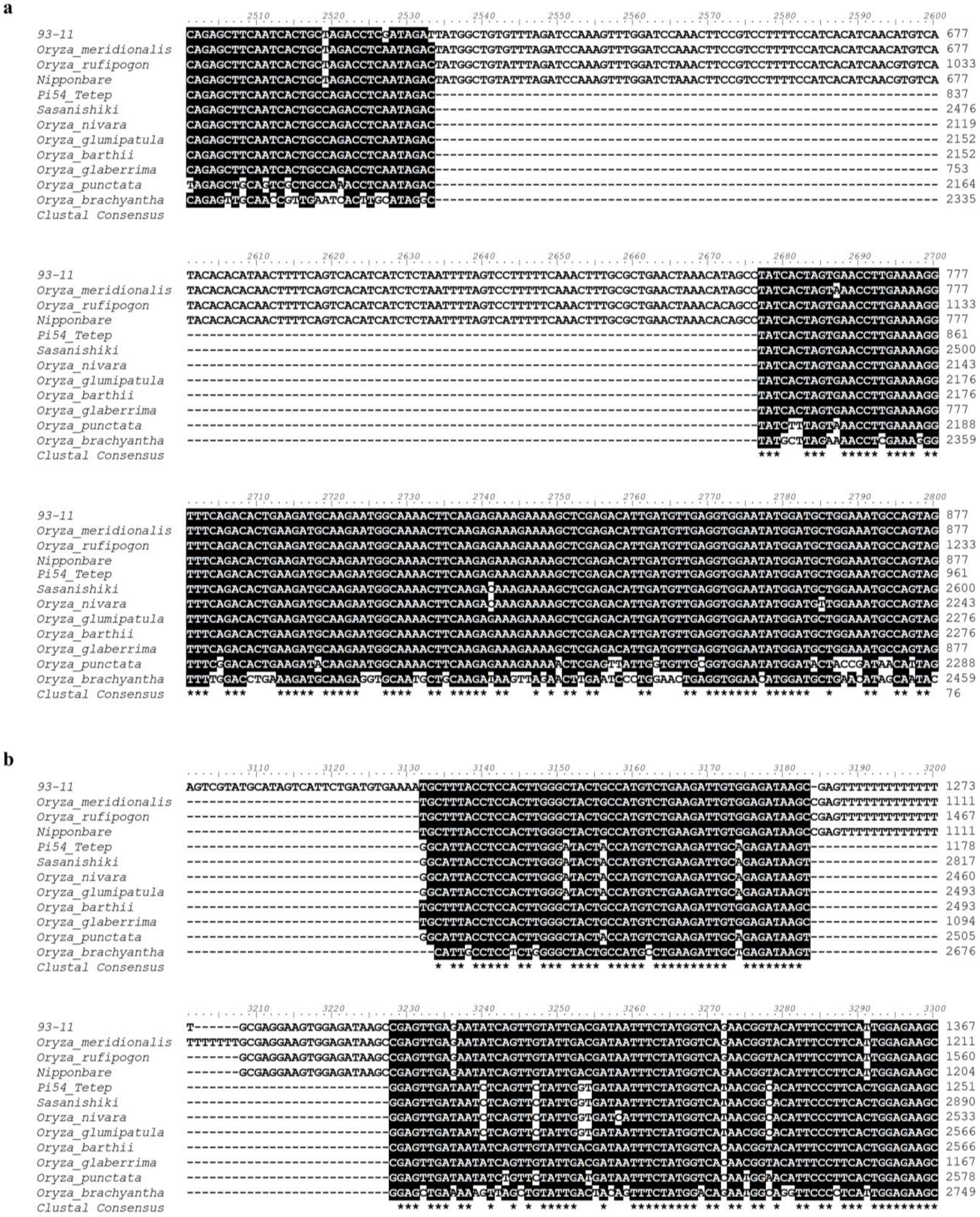

Figure S4 DNA sequences of *Pi54* alleles. (a) A 143 bp insertion in Nipponbare type species. (b) A 37 or 44 bp insertion in Nipponbare tpye species. 93-11 has an additional 31 bp insertion.

```
                              2310      2320      2330      2340      2350
                         ....|....|....|....|....|....|....|....|....|....|
Helitron-N91_OS          CTCAATAGGAAAAACAAAACATAGAGCGAGCTTTTATAGCCTGAGGCTGT 2350
Nipponbare (143 bp)      --------------------------------------------GGCTGT 6
Clustal Consensus                                                    ******

                              2360      2370      2380      2390      2400
                         ....|....|....|....|....|....|....|....|....|....|
Helitron-N91_OS          GTTTAGTTCAGCGCAAAGTTTGGATTTTGGTTGAAATTGGAGATGATGTG 2400
Nipponbare (143 bp)      GTTTAGTTCAGCGCAAAGTTTGAAAAATGACTAAAATTAGAGATGATGTG 56
Clustal Consensus        ********************** *   **  * ***** *********** 48

                              2410      2420      2430      2440      2450
                         ....|....|....|....|....|....|....|....|....|....|
Helitron-N91_OS          ACTGAAAAGTTGTGTGTGTATGACAGGTTGATGTGATGGAAAAGGACTGA 2450
Nipponbare (143 bp)      ACTGAAAAGTTGTGTGTGTATGACACGTTGATGTGATGGAAAAGGACGGA 106
Clustal Consensus        ************************* ********************* ** 96

                              2460      2470      2480      2490      2500
                         ....|....|....|....|....|....|....|....|....|....|
Helitron-N91_OS          AGTTTGGATCCAAACTTTGGATCTAAACACAGCCTAAATGTGAAACTAAT 2500
Nipponbare (143 bp)      AGTTTAGATCCAAACTTTGGATCTAAATACAGCCATA------------- 143
Clustal Consensus        ***** ********************* ******  *              129
```

Figure S5 Alignment of Helitron-N91 and the 143 bp insertion in Nipponbare. The sequence of Helitron-N91, which is a nonautonomous DNA transposon, was downloaded from the Repbase database; its total length is 4,147 bp.

Table S1 Primer sets for PCR amplification

Primer sets	5' – 3'	Position	Length
***Pi54* MAS**	CAATCTCCAAAGTTTTCAGG	25264138	358
	GCTTCAATCACTGCTAGACC	25264495	
**Os11g0638700 (#1)**	CATTGACTCTGGCGTGCTAA	25239450	656
	GCAAAGGCCATACCAAGTGT	25238795	
**Os11g0638900 (#2)**	GGCCTCTTCTGTTCGAGTTG	25246990	608
	TGTCCAAGCAGTGTTCAAGC	25246383	
**Os11g0639000 (#3)**	GAGAAGAGGCTGGTCAAACG	25367562	780
	CAGCCAGCAAAACAAGTTCA	25368341	
**Os11g0639100 (#4, *Pi54*)**	TTAGGCCTTCAGGAATGGAG	25263384	900
	TGCAAGAATGGCAAAACTTC	25264283	
**Os11g0639300 (#5)**	GCAACCTTTCCAAGAAGCAG	25276304	481
	CCGTACTCCTCTCGCTTGAC	25276784	
**Os11g0639400 (#6)**	AAGCGCAAGAAATCGGAGT	25280431	458
	AACGGTGAAAAATCGTCTGG	25279956	
**Os11g0639600 (#7)**	GCAAGCTAAGGGATGATTCG	25290044	877
	CTGGCTACCGACCAACAAAT	25289168	
**Os11g0640000 (#8)**	CCGGTGTCGGAGTACAGATT	25317721	736
	ATCAGCTCGATGTGGCTCTT	25316986	
**Os11g0640300 (#9)**	TGAGCTCGAGCATTTGACAC	25324341	551
	CAACACCTGCCAATCCTTCT	25324891	
**Os11g0640500 (#10)**	GAATCGCGTAGGTCTCCAAG	25334592	496
	CCAAACTACGCCAAATTCGT	25335087	
**Os11g0640600 (#11)**	AGGCTTTCTCGGCTACACAA	25343366	437
	TCATACGCAACAAGCTCCAG	25342930	
**Os11g0640800 (#12)**	TCGAATCGCTCAAGATCAGA	25359846	371
	TTGTCCACGGATGAACTTTG	25360216	
**Os11g0641200 (#13)**	CCGCAGCTCAAATTTCGGAG	25395091	751
	AGGGCAGTGTGATCCAACAG	25395841	
**Os11g0641300 (#14)**	TGCATACCCTCTGCTTCCTT	25399767	1150
	TCCAGGAACGACAAGCAATA	25400916	

Table S2 Primer sets for identification of Nipponbare and Sasanishiki type species

Primer set type	Loci	Primer ID	5'-3'
**Boundary position**	Left	Common_forward	TGGTGTGCGTTTCTCTCCTA
		N_reverse [a]	TTTGACACCTTAGGCCGAGT
		S_reverse [b]	GGAAAGGGAATTGAGCACAG
	Right	Common_reverse	TCAGCTTGGTGAGCTCATTG
		N_ forward	CCCAGAACAGACCCTTGTGT
		S_ forward	CCGAGTGTGCATATTGTCCTT
**Coding region**	*Pi54* of S	S_ forward	AGCTACGATCAAGCCAGCAT
		S_reverse	GGATTGGGAAAACTCGCATA
	#11 of S	S_ forward	CCTGGAATGCCTTGTTGATT
		S_reverse	GAGGGGAATACCTCCTCAGC

[a] N, Nipponbare
[b] S, Sasanishiki

Table S3 Primers for sequencing

**Forward primers**	**5'-3'**	**Reverse primers**	**5'-3'**
pIB FP	GGATGTGCTGCAAGGCGATTAAGTTGG	pIB RP	CTCGTATGTTGTGTGGAATTGTGAGC
5aT5Primer_F	AGATCAGCGGTGGAAAATTG	6bT5Primer_R	TTGTCCACGGATGAACTTTG
5at4	TTAGGCCTTCAGGAATGGAG	6bT5_1R	TCAGCTTGGTGAGCTCATTG
primer MAS-a	CAATCTCCAAAGTTTTCAGG	6bT5_2R	GGATGAGGAGTCGCTTCTTG
Long_seq_1F	GCAGCGCTGATCAGAGAATA	6bT5_3R	CGTCCAGCCACTTCGTCTT
Long_seq_2F	GCCAGCATCTCTTCAACCAT	Long_seq_1R	TCGCAAAGACAAATGAGATG
Long_seq_3F	AACAGTGTGTTGGTCGTTGC	Long_seq_2R	TTGATCTCCGTGGGACACTT
Long_seq_4F-1	CCTCCTGCCCACCTTATATAG	Long_seq_3R	AGTGGCCATTATCCCACAGA
Long_seq_5F	TCCAAAGTATTGGGCATACG	Long_seq_4R	TGCACTCAGTGTATGGAAAGG
Long_seq_6F	TCCTGACTGGCGCAAGTATT	Long_seq_5R	CTGGAAGGCCTTGAAAAGGT
Long_seq_7F	GATGCTCATCAGCGTTTGTG	Long_seq_6R	ATGGGTCTGCAGGATCTAGG
Long_seq_8F	GATGTCCGAATAGAAATCGTGA	Long_seq_7R	TCCAATTCTGCCACACAGAG
Long_seq_9F_1	GCGACGGTACTAGTCAGCAG	Long_seq_9R	TGCGAGCTTCTTCTAACTTCG
Long_seq_10F	AGACGTAGACGCCCAACAAC	Long_seq_10R	TGATGAAGAAACCCACATCG
M13-forward	GTAAAACGACGGCCAGTGAG	Long_seq_11R	GGATTGAAAGCCAAAATAGCC
M13_HL2-8_2F	TCCAATTCTGCCACACAGAG	Long_seq_12R	GTCCCAATCCCTGAAACAGA
M13_HL2-8_3F	ATGCAGTGGAACGGCATTAG	Long_seq_13R	AGCGAGCACTCCTTCCCTAT
M13_HL2-8_4F	AGTGTCATCTGCTGGGGTTG	Long_seq_14R	GTTTCAGGGCCAACTGAACA
		Long_seq_15R	GACAGTTGGTGCGTCAGGTA
		Long_seq_16R	TGGTTGCTTGTAGGCGTTAG
		Long_seq_17R	CTGCCGTCATCAAGAGGAAT
		5bt4	TGCAAGAATGGCAAAACTTC
		M13-reverse	GGAAACAGCTATGACCATG
		M13_HL2-8_2R	GCTCAGTGCTTGTCATCCAA
		M13_HL2-8_3R	CAGCATTGGAATGACCAACT
		M13_HL2-8_4R	TGACGAGCATTCCAAACATT

Table S4 Primers for sequencing the genes flanking the 25,061 bp region in Sasanishiki

Gene ID	Primers	5'-3'
**Os11g0638700 (2,241 bp)**	Os11g0638700_PCR_F	GCTGCACCTCTTCACCCTAC
	Os11g0638700_PCR_R	TCCAACAGAATGGAAGCACA
	Os11g0638700_seq_R1	GCTGAGGATGTGCTCCACTT
	Os11g0638700_seq_R2	CTGGGTTGCTGAGTCCAAGA
	Os11g0638700_seq_R3	ACCACCGTTGCATTCTTTCT
**Os11g0638900 (1,835 bp)**	Os11g0638900_PCR_F	CTTTTGTGGATGGAGGGAGT
	Os11g0638900_PCR_R	TTCTTCAATCTGGGGCTGTC
	Os11g0638900_seq_F	GGTACCTTGCAATCCAAGCA
**Os11g0639000 (4,349 bp)**	Os11g0639000_PCR_F	TATGGGCCTTCTGTTGCTCT
	Os11g0639000_PCR_R	CAACACCTGGCAGTTCTCAC
	Os11g0639000_seq_F1	TTGGGAGATCTAGGGCTACTTTC
	Os11g0639000_seq_F2	GCCATACCAGTGACCCAATC
	Os11g0639000_seq_F3	TTTACTGTTGTCCGCTGCAT
	Os11g0639000_seq_F4	CAAAAGAGGCAAGGTATGGTG
	Os11g0639000_seq_F5	TTGGAAGTTGAGTGCACATTCT
	Os11g0639000_seq_F6	TGGGATTATGTAACCCAGTTGA
**Os11g0641200 (5,361 bp)**	Os11g0641200_PCR_F	TTCGTGGGAATTGAACAGTG
	Os11g0641200_PCR_R	GAGTGACAGGCAGGACAACA
	Os11g0641200_seq_F1	GGAATTGGGGGCAAACTATC
	Os11g0641200_seq_F2	TGCAGGCTTGCTATATTGGAG
	Os11g0641200_seq_F3	GGCCAGAAAGGAAGGGTACT
	Os11g0641200_seq_F4	CTTCCACCTTGTGCTGCTTC
	Os11g0641200_seq_F5	TGTTGCACTCTGGCATTGTT
	Os11g0641200_seq_F6	TGCAGATAGGAGACAAAGCA
	Os11g0641200_seq_F7	TTGCTGAAGGCTGTCCTCTT
**Os11g0641300 (765 bp)**	Os11g0641300_PCR_F	TGCATACCCTCTGCTTCCTT
	Os11g0641300_PCR_R	TCCAGGAACGACAAGCAATA

Accession codes Sequence data of Sasanishiki the *Pi54* locus

Sequence data of Sasanishiki *Pi54* locus have been deposited at DNA Data Bank of Japan (DDBJ) with the following accession number: the region from *Pi54* to #12, LC385788; #1 gene, LC385789; #2 gene, LC385790; #3 gene, LC385791; #13 gene, LC385792; #14 gene, LC385793.

Table S5 Transposable elements in the flanking regions of *Pi54*, #5, #10 and #11

Transposable element	Repeat class/family	*Bra*			Nip			*Ruf*			*Mer*			*Niv*
		#3–#11	#7–#5	#5–#13	#4–#5	#10–#11	#11–#12	#4–#5	#10–#11	#11–#12	#4–#5	#10–#11	#11–#12	#4–#11
ATLANTYS-LTR	LTR/Gypsy					+			+			+		
CLOUD-7	DNA/MULE-MuDR											+		
Copia-19_OS-LTR	LTR/Copia	+												
DITAILA	DNA/PIF-Harbinger				+			+			+			
DITTO	DNA/PIF-Harbinger			+	+			+			+			
DITTO3	DNA/PIF-Harbinger						+			+			+	
DNA9-27	DNA													+
EXPLORER	DNA			+										
EXPLORER1	DNA										+			
GAIJIN	DNA/PIF-Harbinger				+			+			+			
Harbinger-N1	DNA/PIF-Harbinger				+		+	+		+	+		+	
Harbinger-N7C	DNA/PIF-Harbinger				+			+			+			
Harbinger-N9	DNA/PIF-Harbinger			+										
HEARTBLEEDING	DNA/PIF-Harbinger		+											
Helitron-N8C	RC/Helitron				+									
Helitron-N107B	RC/Helitron						+			+				
LTR-17B_OS-LTR	LTR		+											
Mariner-N2	DNA/TcMar-Stowaway				+			+			+			
NDNA1TNA	DNA/PIF-Harbinger			+										
RIREX_LTR	LTR/Gypsy													+
RIREXE_I-int	LTR/Gypsy													+
SNAP-OL3	DNA	+	+											
STOWAWAY14	DNA/TcMar-Stowaway													+
STOWAWAY34	DNA/TcMar-Stowaway						+			+			+	
TOURIST-XI	DNA/PIF-Harbinger													+
TREP215	DNA/TcMar-Stowaway			+										
Total number		2	3	5	7	1	4	6	1	4	7	2	3	5

Table S6 Genes Orthologous to a mobile unit on chromosome 1 in *Oryza* species

O. punctata	*O. brachyantha*	*Nipponbare*	*O. rufipogon*	*O. meridionalis*	*O. nivara*	*O. glumaepatula*
OPUNC01G04000	OB01G13960	Os01g0165600	ORUFI01G04390	OMERI01G04130	ONIVA01G04660	OGLUM01G04590
**OPUNC01G04010** [a]	**OB01G13970**	-	-	-	-	-
OPUNC01G04020	-	-	-	-	-	-
OPUNC01G04030	-	-	-	-	-	-
OPUNC01G04040	-	-	-	-	-	-
**OPUNC01G04050**	**OB01G13970**	-	-	-	-	**OGLUM01G04610**
OPUNC01G04060	-	-	ORUFI01G04400	OMERI01G04150	ONIVA01G04670	OGLUM01G04600

[a] Bold front, genes belong to the #5 gene family
